# The Association of Family History of Premature Cardiovascular Disease or Diabetes Mellitus on the Occurrence of Gestational Hypertensive Disease and Diabetes

**DOI:** 10.1371/journal.pone.0167528

**Published:** 2016-12-05

**Authors:** Dong-Ju Choi, Chang-Hwan Yoon, Heesun Lee, So-Yeon Ahn, Kyung Joon Oh, Hyun-Young Park, Hea Young Lee, Myeong Chan Cho, Ick-Mo Chung, Mi-Seung Shin, Sung-Ji Park, Chi Young Shim, Seong Woo Han, In-Ho Chae

**Affiliations:** 1 Cardiovascular Center, Seoul National University Bundang Hospital, Seongnam, Korea; 2 MRCC, Seoul National University Bundang Hospital, Seongnam, Korea; 3 Department of Obstetrics & Gynecology, Seoul National University Bundang Hospital, Seongnam, Korea; 4 Division of Cardiovascular and Rare Diseases, Center for Biomedical Science, National Institute of Health, Chungbuk, Korea; 5 Korean Nurses Association, Seoul, Korea; 6 Department of Internal Medicine, Chungbuk National University, Chungbuk, Korea; 7 Cardiology Division, School of Medicine, Ewha Womans University, Seoul, Korea; 8 Division of Cardiology, Department of Internal Medicine, Gachon University Gil Medical Center, Incheon, Korea; 9 Division of Cardiology, Cardiovascular Imaging Center, Heart Vascular Stroke Institute, Samsung Medical Center, Sungkyunkwan University School of Medicine, Seoul, Korea; 10 Cardiology Division, Severance Cardiovascular Hospital, Yonsei University, Seoul, Korea; 11 Department of Cardiovascular Medicine, Dongtan Sacred Heart Hospital, Hwaseong, Korea; Jichi Medical University, JAPAN

## Abstract

**Background:**

Gestational hypertensive diseases (GHD) and gestational diabetes mellitus (GDM) increase the risk of cardiovascular disease (CVD) later in life. However, the association between gestational medical diseases and familial history of CVD has not been investigated to date. In the present study, we examined the association between familial history of CVD and GHD or GDM via reliable questionnaires in a large cohort of registered nurses.

**Methods:**

The Korean Nurses’ Survey was conducted through a web-based computer-assisted self-interview, which was developed through consultation with cardiologists, gynecologists, and statisticians. We enrolled a total of 9,989 female registered nurses who reliably answered the questionnaires including family history of premature CVD (FHpCVD), hypertension (FHH), and diabetes mellitus (FHDM) based on their medical knowledge. Either multivariable logistic regression analysis or generalized estimation equation was used to clarify the effect of positive family histories on GHD and GDM in subjects or at each repeated pregnancy in an individual.

**Results:**

In this survey, 3,695 subjects had at least 1 pregnancy and 8,783 cumulative pregnancies. Among them, 247 interviewees (6.3%) experienced GHD and 120 (3.1%) experienced GDM. In a multivariable analysis adjusted for age, obstetric, and gynecologic variables, age at the first pregnancy over 35 years (adjusted OR 1.61, 95% CI 1.02–2.43) and FHpCVD (adjusted OR 1.60, 95% CI 1.16–2.22) were risk factors for GHD in individuals, whereas FHH was not. FHDM and history of infertility therapy were risk factors for GDM in individuals (adjusted OR 2.68, 95% CI 1.86–3.86; 1.84, 95% CI 1.05–3.23, respectively). In any repeated pregnancies in an individual, age at the current pregnancy and at the first pregnancy, and FHpCVD were risk factors for GHD, while age at the current pregnancy, history of infertility therapy, and FHDM were risk factors for GDM.

**Conclusions:**

The FHpCVD and FHDM are significantly associated with GHD and GDM, respectively. Meticulous family histories should be obtained, and women with family histories of these conditions should be carefully monitored during pregnancy.

## Background

Gestational diabetes mellitus (GDM) and gestational hypertensive disorders (GHD), comprising preeclampsia and gestational hypertension, are common medical complications in pregnancy that result in high maternal mortality and morbidity and poorer pregnancy outcomes.[1, 2] They are also well-known risk factors for cardiovascular disease (CVD) later in maternal life.[3–9] However, it remains unclear whether GHD and GDM initiate vascular changes that become clinically evident later or whether a pre-pregnancy predisposition to CVD increases the risk of GHD and GDM. GHD and GDM may induce cardiovascular change by promoting endothelial inflammation and early atherosclerosis, independent of underlying conditions.[10–12] Conversely, GHD and GDM may be a result of inherent susceptibility to CVD including genetic factors. Mothers who were obese or had a personal history of chronic hypertension or diabetes before pregnancy were more likely to develop GHD or GDM.[13–15] Furthermore, family history of cardiovascular risk factors has been well established to closely relate to the occurrence of future CVD.[16, 17] To the best of our knowledge, no previous report have evaluated the association between gestational medical diseases and familial history of cardiovascular disease such as stroke, angina, or myocardial infarction. In the present study, we investigated the association between familial history of cardiovascular disease and GHD or GDM via reliable questionnaires in a large cohort of registered nurses.

## Methods

### Study population

**Fig 1:**
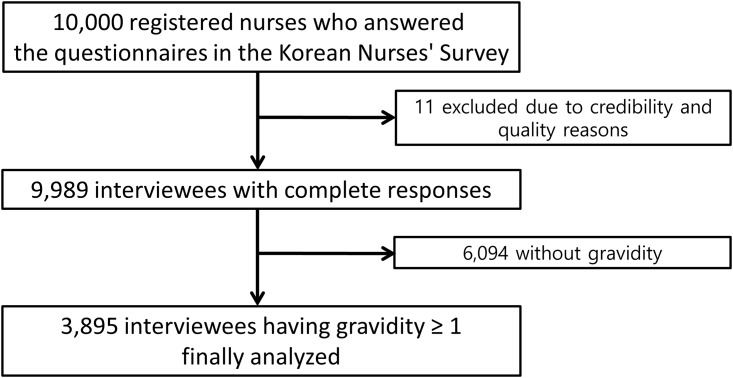
Study flow.

The Korean Nurses’ Survey is a cross-sectional survey that was conducted through web-based computer-assisted self-interview (CASI) from October to December 2011 for the purpose of assessing the association between lifestyle and CVD in Korean women to identify targets for preventive measures in this population. The Korean National Institute of Health performed this survey in collaboration with the Korean Nurses Association and Seoul National University Bundang Hospital. The number of registered nurses in South Korea is estimated to be 250,000. The Korean Nurses Association sent all registered nurses currently licensed to practice in Korea e-mails containing a link to the web-based CASI and advertised the survey on its web page, which also linked to the CASI. Among them, 10,000 nurses registered on the website, and six who were younger than 20 years of age, one who was not identified in the nurse registry, and four with incomplete responses were excluded from the study. We finalized the database with a total of 9,989 female registered nurses, who reliably answered the questionnaires based on their medical knowledge. Among them, 3,895 subjects with a history of at least one pregnancy were included and analyzed in the present study (Fig 1). The study protocol was approved by the Institutional Review Board of Seoul National University Bundang Hospital (IRB No. B-1111-139-012). Electronic informed consent was obtained from all study participants which was approved by the IRB.

### Questionnaires

The questionnaires had 175 questions that were compiled by cardiologists, gynecologists, endocrinologists, and statisticians, and it collected information regarding demographic characteristics; comorbidities; past medical history and current medications; family history of diseases; obstetrical, social, psychiatric and occupational characteristics; and dietary patterns (S1 and S2 Files). Next, we developed a CASI system that produced a database at the same time as an interviewee answered the questions on the web-based program. We included a function that automatically skipped unnecessary questions to reduce the total response time for 175 questions, which was 19 min on average for the 100 pilot interviewees. The reliability and validity of the questionnaire was tested in 100 subjects with offline interviews. We compared the data including height, weight, and waist circumference from CASI system and offline interviews. There was no significant difference between the data.

The questionnaire collected information including demographic data, underlying diseases, cardiovascular symptoms, history of cardiovascular diseases, menarche, menstruation, menopause, history of contraception, infertility therapy, smoking, exercise, and dietary records. Family history of cardiovascular diseases and risk factors were also evaluated on the questionnaire. The family history included 1) whether first-degree blood relatives of the interviewee had a premature cerebral or cardiac vascular disease (CVD), which was defined as CVD diagnosed before 55 years of age in men or 65 years of age in women[18], or whether first-degree blood relatives of the interviewee had the following diseases at any age: 2) hypertension, 3) dyslipidemia, 3) diabetes mellitus, 4) stroke, 5) angina, or 6) myocardial infarction. Gestational data were also obtained from the questionnaire, including the number of times an interviewee became pregnant (gravidity), gave birth to a baby (parity), and the details of each pregnancy (up to 5) including age at the pregnancy, duration, outcome, whether the interviewee experienced GHD or GDM, delivery method, birth weight, breast feeding duration, and postpregnancy weight gain.

### Statistical analysis

Demographic and obstetric characteristics and familial cardiovascular risk factors were compared according to interviewees’ experience of GHD or GDM. Continuous variables are expressed as mean ± standard deviation (SD), whereas categorical variables are presented as absolute values and proportions. Differences between continuous variables were compared using the Student *t* test for independent samples, and differences between categorical variables were compared using the *χ*^2^ test or Fisher exact test, as appropriate. Multivariable logistic regression analysis was conducted to assess the association between family history of cardiovascular risk factors and gestational medical diseases and expressed with an odds ratio (OR) and corresponding 95% confidence interval (CI) after adjusting for potential confounders. We also analyzed the associations at each pregnancy, which included multiple pregnancies of a subject using generalized estimating equations because every pregnancy had potential confounding factors including age at the pregnancy and the order of pregnancy (primi-para vs. multi-para). A value of *p* <0.05 was considered to indicate statistical significance. All analyses were performed using SPSS 21.0 statistical software (SPSS Inc., IBM Co., Chicago, IL, USA).

## Results

### Baseline demographic, obstetric, and gynecologic characteristics

**Fig 2:**
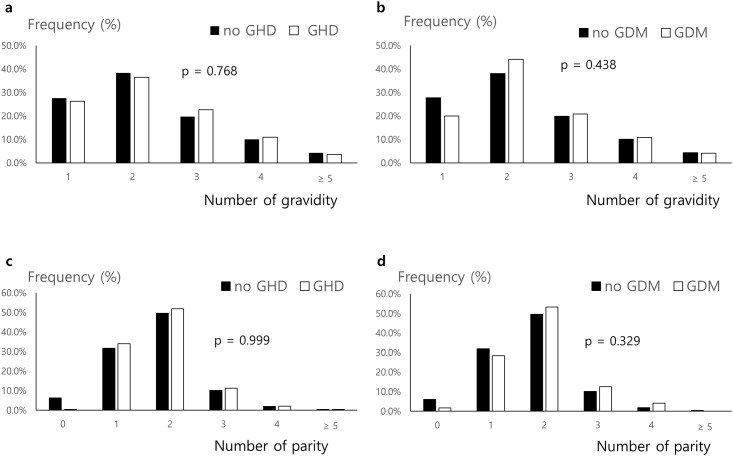
Gravidity and parity of the study subjects. (A) Histogram of the number of gravidity in subjects with GHD or no GHD. (B) Histogram of the number of gravidity in subjects with GDM or no GDM. (C) Histogram of the number of parity in subjects with GHD or no GHD. (D) Histogram of the number of parity in subjects with GDM or no GDM.

**Fig 3:**
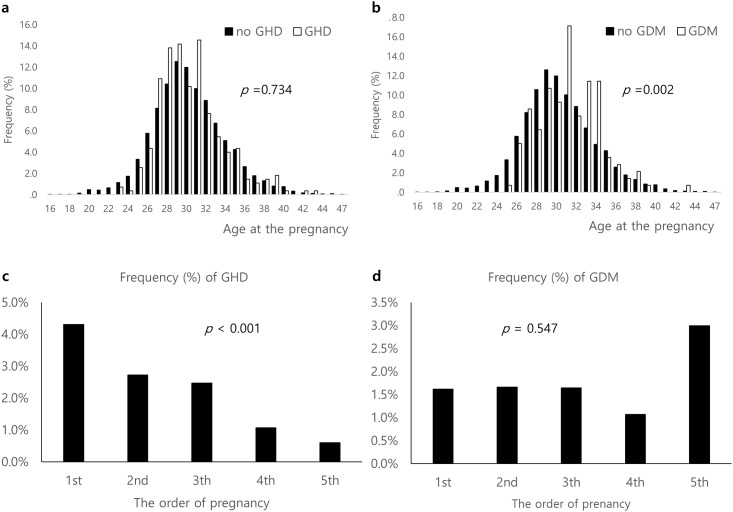
Distribution of age at every repeated pregnancy and frequency of GHD or GDM according to the order of pregnancy. (A) Histogram of age at the pregnancy in subjects with GHD or no GHD. (B) Histogram of age at the pregnancy in subjects with GDM or no GDM. (C) Incidence of GHD according to the order of pregnancy. (D) Incidence of GDM according to the order of pregnancy.

In the Korea Nurses’ Survey, a total of 3,895 study participants (age 40.3 ± 8.0 years; age at the 1st pregnancy 28.6 ± 3.2 years) had at least one pregnancy, cumulatively reporting a total of 8,783 pregnancies and 6,685 parities (Table 1). Among them, 247 interviewees (6.3%) responded that they had experienced GHD in at least one pregnancy, which included preeclampsia (n = 120, 3.1%) and transient hypertension (n = 127, 3.2%), and 120 (3.1%) responded that they had experienced GDM in at least one pregnancy. Interviewees with a history of GHD had higher BMI at the time of survey completion, whereas those with a history of GDM did not. Gravidity and parity were not different between the no-GHD and GHD groups or the no-GDM and GDM groups (Fig 2). Those with GHD or GDM were older at the first pregnancy as well as at the first parity. The age distribution and average age at each pregnancy among the 8,783 cumulative pregnancies did not differ between pregnancies involving GHD and other pregnancies (Fig 3A). However, the average age at pregnancies involving GDM was higher than of pregnancies without GDM (Fig 3B). The prevalence of GHD gradually decreased with progressive pregnancies, whereas GDM prevalence did not differ according to the order of pregnancies (Fig 3C and 3D).

**Table 1:** Baseline characteristics of the study population.

	Total (n = 3,895)	GHD (+) (n = 247)	GHD (-) (n = 3,648)	*p*	GDM (+) (n = 120)	GDM (-) (n = 3775)	*p*
Age at the survey (years)	40.3 ± 8.0	40.9 ± 7.9	40.3 ± 8.0	0.241	39.9 ± 6.5	40.4 ± 8.0	0.467
BMI (kg/m^2^) at the survey	21.8 ± 2.7	22.4 ± 2.5	21.8 ± 2.5	< 0.001	22.1 ± 2.4	21.9 ± 2.6	0.299
Age at first pregnancy	28.6 ± 3.2	29.0 ± 3.1	28.6 ± 3.3	0.046	29.3 ± 2.9	28.6 ± 3.3	0.032
Age at first parity	28.5 ± 3.1	29.4 ± 3.0	28.9 ± 3.0	0.052	29.6 ± 2.9	29.0 ± 3.0	0.025
Age at first event		30.0 ± 3.3			31.1 ± 3.2		
Menarche ≤ 12years	598 (15.4%)	42 (17%)	556 (15.2%)	0.465	19 (15.8%)	579 (15.3%)	0.898
Menstrual regularity (<4 days)	2492 (64%)	167 (67.6%)	2325 (63.7%)	0.244	67 (55.8%)	2425 (64.2%)	0.066
Menstrual cycle (<30 days)	3015 (77.4%)	201 (81.4%)	2814 (77.1%)	0.135	82 (68.3%)	2933 (77.7%)	0.020
Use of oral contraceptive pill	355 (9.6%)	26 (9.6%)	329 (9.6%)	0.956	12 (10.0%)	361(9.6%)	0.873
Infertility therapy	295 (7.6%)	22 (8.9%)	273 (7.5%)	0.778	15 (12.5%)	280 (7.4%)	0.038

GHD = gestational hypertensive disease; GDM = gestational diabetes mellitus; BMI = body mass index.

**Fig 4:**
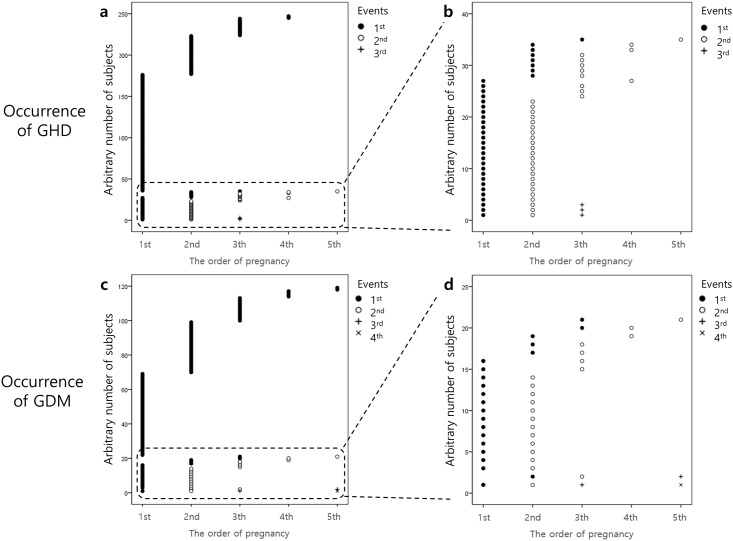
Distribution of the occurrence of GHD or GDM according to the order of pregnancy. (A) The x-axis denotes the order of pregnancy. The y-axis indicates the arbitrary numbers of subjects. Every number matches each patient with GHD. To better demonstrate the distribution of the occurrence of GHD, subjects with more occurrences and subjects with earlier occurrence were placed at lower arbitrary numbers. (B) A magnified graph from the dashed-line rectangle in (A). (C), (D) Similarly, distribution of the occurrence of GDM according to the order of pregnancy.

Among 247 patients with GHD, 35 had at least two events of GHD in sequential pregnancies. Among 120 patients with GDM, 21 had at least two events of GDM in sequential pregnancies. The distributions of the initial occurrence of GHD or GDM and their recurrence according to the order of pregnancy are shown in Fig 4. The rates of GHD or GDM recurrence at the subsequent pregnancy in the subjects who experienced GHD or GDM at the previous pregnancy were 22.3% (23 of 103) and 33.3% (13 of 39), respectively. The GDM group reported more frequent menstrual irregularity and longer menstrual cycles and had a higher rate of infertility therapy than the no-GDM group (Table 1). Twenty-five interviewees (0.6%) responded that they had experienced both GHD and GDM. Twenty-two patients experienced both GHD and GDM in the same pregnancy. Their characteristics were not noticeable compared to those with GHD or GDM (S1 Table).

### Familial history of cardiovascular risk factors and diseases

The familial history of CVD had different patterns in the GHD and GDM groups (Table 2). Subjects with GHD had more frequent positive familial histories of premature CVD (FHpCVD), hypertension (FHH), dyslipidemia, and stroke than those without GHD. However, those with GDM showed more frequent familial history of DM (FHDM) and angina pectoris. The detailed prevalence of familial history of cardiovascular risk factors and diseases is shown in Table 2.

**Table 2:** Familial history of cardiovascular risk factors and diseases.

	Total (n = 3,895)	GHD (+) (n = 247)	GHD (-) (n = 3,648)	*p*	GDM (+) (n = 120)	GDM (-) (n = 3775)	*p*
Premature CVD*	955 (24.5%)	85 (34.4%)	870 (23.8%)	< 0.001	37 (30.8%)	918 (24.3%)	0.106
Hypertension	2,245 (57.6%)	161 (65.2%)	2,084 (57.1%)	0.014	75 (62.5%)	2,170 (57.5%)	0.302
Diabetes mellitus	1,138 (29.2%)	75 (30.4%)	1,063 (29.1%)	0.665	62 (51.7%)	1,076 (28.5%)	< 0.001
Dyslipidemia	1,226 (31.5%)	92 (37.2%)	1,134 (31.1%)	0.047	46 (38.3%)	1,180 (31.3%)	0.110
Stroke	608 (15.6%)	55 (22.3%)	553 (15.2%)	0.005	24 (20.0%)	584 (15.5%)	0.200
Angina pectoris	352 (9.0%)	23 (9.3%)	329 (9.0%)	0.819	19 (15.8%)	333 (8.8%)	0.014
Myocardial infarction	193 (5.0%)	12 (4.9%)	181 (5.0%)	0.942	4 (7.3%)	189 (4.6%)	0.524

* Premature CVD was defined as CVD diagnosed before 55 years of age in men or 65 years of age in women.

CVD = cardiovascular disease; GHD = gestational hypertensive disease; GDM = gestational diabetes mellitus.

### The association of familial history of cardiovascular risk factors and diseases and the occurrence of GHD or GDM in subjects

Among demographic, obstetric, and gynecologic variables, the occurrence of GHD in an individual was closely related to age at the first pregnancy ≥ 35 years and family history of premature CVD, hypertension, dyslipidemia, or stroke in univariable analyses (Table 3). In multivariable analysis, adjusting for the significant variables in univariable analyses as well as obstetric and gynecologic variables, age and FHpCVD were associated with the occurrence of GHD in a subject (adjusted OR [95% CI] 1.61 [1.02–2.43]; 1.60 [1.16–2.22], respectively). The occurrence of GDM was associated with a longer menstrual cycle (>30 days), history of previous infertility therapy, FHDM, and family history of angina pectoris in univariable analyses. In multivariable analysis, history of previous infertility therapy, FHDM, and family history of angina were risk factors for the occurrence of GDM in a subject (adjusted OR 2.68, 95% CI 1.86–3.86; 1.79, 95% CI 1.00–3.01).

**Table 3:** Univariable and multivariable analysis of risk factors for GHD or GDM in subjects.

**A. GHD**	**Unadjusted OR (95% CI)**	Adjusted OR* **(95% CI)**
Age at first pregnancy ≥ 35 years	1.63 (1.04–2.45)	1.61 (1.02–2.43)
Familial history of		
Premature CVD	1.68 (1.27–2.20)	1.60 (1.16–2.22)
Hypertension	1.40 (1.07–1.84)	
Diabetes mellitus	1.06 (0.80–1.40)	
Dyslipidemia	1.32 (1.01–1.72)	
Stroke	1.60 (1.16–2.18)	
Angina pectoris	1.04 (0.67–1.62)	
Myocardial infarction	0.98 (0.54–1.78)	
**B. GDM**	**Unadjusted OR (95% CI)**	Adjusted OR* **(95% CI)**
Age at first pregnancy ≥ 35 years	1.43 (0.74–2.53)	
Menstrual regularity (<4 days)	0.70 (0.49–1.02)	
Menstrual cycle (<30 days)	0.62 (0.42–0.93)	
Infertility therapy	1.78 (1.02–3.11)	1.84 (1.05–3.23)
Familial history of		
Premature CVD	1.39 (0.94–2.06)	
Hypertension	1.23 (0.85–1.79)	
Diabetes mellitus	2.68 (1.86–3.86)	2.68 (1.86–3.86)
Dyslipidemia	1.37 (0.94–1.99)	
Stroke	1.37 (0.87–2.16)	
Angina pectoris	1.94 (1.18–3.22)	1.79 (1.00–3.01)
Myocardial infarction	0.65 (0.24–1.79)	

* Adjusted by age at first pregnancy ≥ 35 years, menarche ≤ 12 years, menstrual regularity (<4 days), menstrual cycle (<30 days), history of infertility therapy, history of oral contraceptive pill, familial history of premature CVD, hypertension, diabetes mellitus, dyslipidemia, stroke, angina pectoris, and myocardial infarction.

CVD = cardiovascular disease; GHD = gestational hypertensive disease; GDM = gestational diabetes mellitus; OR = odds ratio.

### The association of familial history of cardiovascular risk factors and diseases and the occurrence of GHD or GDM at the first pregnancy ending in delivery in subjects

The first pregnancy ending in delivery is a higher-risk pregnancy involving more frequent pregnancy-related complications including GHD and GDM. Therefore, the impact of familial history on the occurrence of GHD and GDM at the first pregnancy ending in delivery is of particular interest. The occurrence of GHD at the first pregnancy ending in delivery was closely related to age at the pregnancy, FHpCVD, FHH, and family history of stroke in univariable analyses (Table 4). In multivariable analysis, age at the pregnancy and FHpCVD were risk factors for the occurrence of GHD in a subject (adjusted OR [95% CI] 1.09 [1.03–1.16]; 1.60 [1.00–2.52], respectively]. The occurrence of GDM was significantly associated with history of previous infertility therapy, FHDM, and family history of dyslipidemia in univariable analyses. In multivariable analysis, age at the pregnancy and FHDM were risk factors for the occurrence of GDM at the first pregnancy ending in delivery (adjusted OR 1.12, 95% CI 1.04–1.20; 2.94, 95% CI 1.81–4.80).

**Table 4:** Univariable and multivariable analysis of risk factors for GHD or GDM at the first parity in subjects.

**A. GHD**	**Unadjusted OR (95% CI)**	Adjusted OR* **(95% CI)**
Age at the pregnancy (y)	1.10 (1.03–1.16)	1.09 (1.03–1.16)
Familial history of		
Premature CVD	1.67 (1.13–2.45)	1.60 (1.00–2.52)
Hypertension	1.47 (1.00–2.19)	
Diabetes mellitus	1.03 (0.68–1.52)	
Dyslipidemia	1.32 (0.90–1.92)	
Stroke	1.77 (1.14–2.69)	
Angina pectoris	0.64 (0.27–1.28)	
Myocardial infarction	0.68 (0.21–1.65)	
**B. GDM**	**Unadjusted OR (95% CI)**	Adjusted OR* **(95% CI)**
Age at the pregnancy (y)	1.13 (1.05–1.21)	1.12 (1.04–1.20)
Menstrual regularity (<4 days)	0.71 (0.45–1.13)	
Menstrual cycle (<30 days)	0.64 (0.39–1.06)	
Infertility therapy	2.47 (1.32–4.64)	
Familial history of		
Premature CVD	0.97 (0.55–1.63)	
Hypertension	1.14 (0.72–1.84)	
Diabetes mellitus	2.90 (1.83–4.62)	2.94 (1.81–4.80)
Dyslipidemia	1.66 (1.03–2.62)	
Stroke	1.01 (0.52–1.82)	
Angina pectoris	0.89 (0.34–1.90)	
Myocardial infarction	0.26 (0.01–1.19)	

* Adjusted by age at the first parity, menarche ≤ 12 years, menstrual regularity (<4 days), menstrual cycle (<30 days), history of infertility therapy, history of oral contraceptive pill, familial history of premature CVD, hypertension, diabetes mellitus, dyslipidemia, stroke, angina pectoris, and myocardial infarction

CVD = cardiovascular disease; GHD = gestational hypertensive disease; GDM = gestational diabetes mellitus; OR = odds ratio.

### Univariable and multivariable analysis of risk factors for GHD or GDM at each repeated pregnancy

We further investigated risk factors for the occurrence of GHD or GDM at each pregnancy including analysis of repeated pregnancies (Table 5). Compared to the first pregnancy, the risk of GHD gradually decreased as the order of pregnancy increased. There was no demographic, obstetric, or gynecologic variable that affected the occurrence of GHD at each pregnancy. FHpCVD, FHH, and family history of stroke were closely related to GHD at each pregnancy in univariable analyses. In multivariable analysis, age, the order of pregnancy, and FHpCVD were risk factors. Interestingly, age at pregnancy was not a risk factor in a univariable analysis, whereas it was a risk factor for the occurrence of GHD in the multivariable analysis.

**Table 5:** Univariable and multivariable analysis of risk factors for GHD or GDM at each pregnancy (total 8,783 pregnancies).

**A. GHD**	**Unadjusted OR (95% CI)**	Adjusted OR* **(95% CI)**
Age at the pregnancy (y)	1.01 (0.98–1.03)	1.06 (1.02–1.09)
The order of pregnancy compared to the first one		
Second pregnancy	0.62 (0.48–0.80)	0.53 (0.40–0.69)
Third pregnancy	0.56 (0.39–0.81)	0.43 (0.28–0.65)
Fourth pregnancy	0.24 (0.11–0.54)	0.19 (0.09–0.43)
≥Fifth pregnancy	0.13 (0.02–0.96)	0.10 (0.01–0.43)
Familial history of		
Premature CVD	1.56 (1.18–2.07)	1.56 (1.13–2.14)
Hypertension	1.34 (1.01–1.76)	
Diabetes mellitus	0.98 (0.73–1.31)	
Dyslipidemia	1.26 (0.96–1.66)	
Stroke	1.39 (1.01–1.92)	
Angina pectoris	1.06 (0.66–1.68)	
Myocardial infarction	0.87 (0.47–1.62)	
**B. GDM**	**Unadjusted OR (95% CI)**	Adjusted OR* **(95% CI)**
Age at the pregnancy	1.07 (1.03–1.10)	1.08 (1.03–1.12)
The order of pregnancy compared to the first one		
Second pregnancy	1.03 (0.74–1.44)	
Third pregnancy	1.02 (0.63–1.64)	
Fourth pregnancy	0.66 (0.28–1.53)	
≥Fifth pregnancy	1.88 (0.76–4.67)	
Menstrual regularity (<4 days)	0.75 (0.51–1.11)	
Menstrual cycle (<30 days)	0.65 (0.43–0.97)	
Infertility therapy	1.89 (1.07–3.33)	1.78 (1.00–3.15)
Familial history of		
Premature CVD	0.64 (0.73–1.65)	
Hypertension	1.10 (0.74–1.63)	
Diabetes mellitus	2.58 (1.75–3.79)	2.62 (1.66–415)
Dyslipidemia	1.39 (0.93–2.07)	
Stroke	1.18 (0.74–1.90)	
Angina pectoris	1.38 (0.84–2.27)	
Myocardial infarction	0.45 (0.17–1.22)	0.35 (0.13–0.94)

* Adjusted by age at the pregnancy, the order of pregnancy compared to the first one, menarche ≤ 12 years, menstrual regularity (<4 days), menstrual cycle (<30 days), history of infertility therapy, history of oral contraceptive pill, familial history of premature CVD, hypertension, diabetes mellitus, dyslipidemia, stroke, angina pectoris, and myocardial infarction.

CVD = cardiovascular disease; GHD = gestational hypertensive disease; GDM = gestational diabetes mellitus; OR = odds ratio.

The age at the pregnancy, a longer menstrual cycle (>30 days), history of infertility therapy, and FHDM were risk factors for GDM in univariable analysis. In GDM analyses, the order of pregnancy was not a risk factor for the occurrence of GDM at each pregnancy. In multivariable analysis, age at the pregnancy, history of infertility therapy, FHDM, and family history of myocardial infarction were significantly associated with the occurrence of GDM.

### Pregnancy outcome after GHD or GDM at each parity

The occurrence of GHD or GDM was associated with different pregnancy outcomes at each pregnancy (Table 6). Pregnancies affected by GHD had higher rates of preterm birth and cesarean section, lower birth weight, greater postpregnancy weight gain, and shorter breastfeeding duration than pregnancies without GHD. Pregnancies affected by GDM had higher rates of full-term birth, cesarean section, higher birth weight, greater post-pregnancy weight gain, and longer breastfeeding duration than those without GDM.

**Table 6:** Pregnancy outcome after GHD or GDM at each parity (total 6,685 parity).

	Total (n = 6,685)	GHD (+) (n = 280)	GHD (-) (n = 6,405))	*p*	GDM (+) (n = 140)	GDM (-) (n = 6,545)	*p*
**Pregnancy duration**				< 0.001			0.002
21–35 wk	450 (5.1%)	47 (16.8%)	403 (6.3%)		9 (6.4%)	441 (6.7%)	
36–41 wk	5,697 (64.9%)	233 (83.2%)	5,464 (85.3%)		131 (93.6%)	5,566 (85.0%)	
≥42 wk	538 (6.1%)	0 (0%)	538 (8.4%)		0 (0%)	538 (8.2%)	
**Delivery method**				< 0.001			<0.001
Vaginal delivery	3,889 (64.0%)	132 (47.7%)	3,757 (64.7%)		60 (43.5%)	3,829 (64.4%)	
Induced vaginal delivery	360 (5.9%)	24 (8.7%)	336 (5.8%)		7 (5.1%)	353 (5.9%)	
Cesarean section	1,832 (30.1%)	121 (43.7%)	1,711 (29.5%)		71 (51.4%)	1,761 (29.6%)	
**Birth weight**				<0.001			<0.001
< 2.5 kg	279 (4.5%)	54 (19.3%)	225 (3.8%)		9 (6.4%)	270 (4.5%)	
2.5–3.5 kg	4,213 (68.5%)	170 (60.7%)	4,043 (68.9%)		67 (47.9%)	4,146 (69%)	
3.5–4.5 kg	1,628 (26.5%)	53 (18.9%)	1,575 (26.8%)		61 (43.6%)	1,567 (26.1%)	
>4.5 kg	27 (0.4%)	3 (1.1%)	24 (0.4%)		3 (2.1%)	24 (0.4%)	
**Postpregnancy weight gain**				0.012			0.047
≤2 kg	2,323 (34.7%)	72 (25.7%)	2,251 (35.1%)		42 (30.0%)	2,281 (34.9%)	
3–5 kg	1,980 (29.6%)	91 (32.5%)	1,889 (29.5%)		33 (23.6%)	1,947 (29.7%)	
6–10 kg	1,630 (24.4%)	82 (29.3%)	1,548 (24.2%)		42 (30.0%)	1,588 (24.3%)	
≥11 kg	752 (11.2%)	35 (12.5%)	717 (11.2%)		23 (16.4%)	729 (11.1%)	
**Breastfeeding duration**				0.070			0.028
≤1 months	2,327 (37.9%)	112 (40.0%)	2,215 (37.8%)		37 (26.4%)	2,290 (38.1%)	
2–3 months	2,080 (33.8%)	91 (32.5%)	1,989 (33.9%)		53 (37.9%)	2,027 (33.7%)	
4–11 months	1,166 (19.0%)	62 (22.1%)	1,104 (18.8%)		36 (25.7%)	1,130 (18.8%)	
≥12 months	574 (9.3%)	15 (5.4%)	559 (9.5%)		14 (10.0%)	560 (9.3%)	

## Discussion

Using the database of an online survey recruiting registered nurses, we found that familial history of cardiovascular diseases is a risk factor for GHD or GDM. FHpCVD was an important risk factor for GHD in individual women or at any pregnancy including repeated pregnancies. At the same time, FHDM was a risk factor for GDM in individual pregnant women or at any pregnancy.

### The advantage of nurse studies

Health professionals, and particularly nurses, have often been selected as study populations for large epidemiological surveys because of their high response rates and high accuracy rates in completing questionnaires.[19, 20] Many investigators have explored associations between cardiovascular risk factors and diseases using the database from the Nurses’ Health Study.[20–22] However, many early CVD prevention trials did not focus on specifically on women and did not include a full range of cardiovascular variables that are specific to women. The investigation of women-specific risk factors using a large survey of nurses is valuable to amassing a more detailed range of evidence to develop recommendations that address specific considerations of women that current guidelines lack.[23]

### GHD and its risk factors

GHD was reported to occur in 5.22% of all pregnancies in a recent study,[4] which is consistent with the prevalence of GHD (6.3%) in the present study. Known GHD risk factors include twin pregnancy, age >35 years, overweight and obesity, primiparity, hypertension, diabetes and FHH. The association of hypertensive disorders of pregnancy with higher offspring blood pressure strongly suggests a genetic link of the diseases.[15] In the present study, we found a significant association between GHD and FHpCVD after multivariable adjustment, whereas FHH lost significance after adjustment. The reason underlying our finding that age at pregnancy was not a risk factor for the occurrence of a GHD in univariable analysis whereas it was a risk factor in multivariable analysis is that GHD occurred mostly at the first pregnancy, and the effect of order of pregnancy was large. Therefore, the effect of age at pregnancy was obscured in univariable analysis. It is evidence that the older a pregnant woman is, the more frequently GHD occurred.

### GDM and its risk factors

The prevalence of GDM varies among populations, similar to the variation of type 2 diabetes, with recent prevalence estimates ranging from 2.8% of pregnant women in Washington, DC, to 18.9% in India and 22% in Sardinia, Italy.[24] There is evidence that old age, obesity, parity, smoking, and family history are risk factors for GDM.[25] Using a computerized database of almost one million births in Australia, researchers also identified old maternal age as well as low socioeconomic position and south Asian ethnicity as important correlates of GDM.[1] We found that old age and FHDM were risk factors for GDM, but parity was not. GDM incidence was sustained over successive pregnancies in contrast to GHD.

### Poor pregnancy outcome: a future risk of cardiovascular disease

In the present study, the subjects who experienced GHD or GDM showed poor pregnancy outcomes such as higher rates of delivering a baby with lower birth weight, preterm delivery, and cesarean section. Pregnancy complications are known to be associated with maternal risk of ischemic heart disease-related hospital admission or death in the future.[5] Therefore, common genetic factors might link family history, gestational disease, and future ischemic heart diseases in the women.

FHpCVD, which was a particularly strong risk factor of GHD in the present study, is a well-known risk factor of coronary heart disease.[18] Therefore, women should be screened for FHpCVD, and then those with a positive FHpCVD and gestational problems should be properly managed to prevent future events. Population-wide studies are also necessary to understand the women-specific risks of CVD, because individual trials including both sexes are likely insufficient to capture and analyze the risks of CVD specific to women such as gestational complications.[26] Understanding sex-based disparities in CVD risks must become an integral strategy to improve cardiovascular outcomes among women and thus reduce the global burden of CVD.

### Limitation

We used a self-reporting questionnaire to assess the association of GHD or GDM with family history. Self-reporting is a valuable epidemiologic tool, but it is known to require additional documentation when the disease is diagnostically complex, for example in myocardial infarction or stroke.[27] We asked the subjects with the following questions: “For pregnancies lasting 20+ weeks, 1) did you have gestational diabetes? 2) did you have gestational hypertension? 3) did you have pre-eclampsia?”. Therefore, diagnosis is largely dependent on the subject’s knowledge of the definition without clear diagnostic criteria. Therefore, this study has a potential bias related to the diagnostic confirmation of the diseases, although we validated several responses from the self-report by off-line interviews and measurements.

This is a retrospective study, which is also affected by recall and selection bias. However, we believe this study to be reliable because several well-established risk factors reported by previous studies were also strong predictors of GHD, GDM, or pregnancy outcomes in the present study, and the subjects were all volunteer registered nurses with an interest in the present study.

All the risk factors were assessed retrospectively and we did obtain any information about whether family history occurred before the GHD or GDM. Therefore, our analyses for risk factors for GHD or GDM may have residual confounding.

## Conclusions

Family history of cardiovascular risk factors was strongly associated with gestational medical disorders such as GHD and GDM in the Korean Nurses’ Survey. Although current guidelines do not recommend the routine evaluation of familial history of cardiovascular risk factors on prenatal testing, meticulous history taking can provide information regarding risks for GHD and GDM.

## Supporting Information

S1 File Questionnaire in English. (DOCX)

S2 File Questionnaire in Korean. (DOCX)

S1 Table Comparison of the subjects with GHD and GDM in the same pregnancy to the others. (DOCX)

## References

[1] Anna V, van der Ploeg HP, Cheung NW, Huxley RR, Bauman AE. Sociodemographic correlates of the increasing trend in prevalence of gestational diabetes mellitus in a large population of women between 1995 and 2005. Diabetes Care. 2008;31(12):2288–93. 10.2337/dc08-1038 18809630 PMC2584183

[2] Ananth CV, Keyes KM, Wapner RJ. Pre-eclampsia rates in the United States, 1980–2010: age-period-cohort analysis. BMJ. 2013;347:f6564 10.1136/bmj.f6564 24201165 PMC3898425

[3] Chambers JC, Fusi L, Malik IS, Haskard DO, De Swiet M, Kooner JS. Association of maternal endothelial dysfunction with preeclampsia. JAMA. 2001;285(12):1607–12. 11268269 10.1001/jama.285.12.1607

[4] Ye C, Ruan Y, Zou L, Li G, Li C, Chen Y, et al The 2011 survey on hypertensive disorders of pregnancy (HDP) in China: prevalence, risk factors, complications, pregnancy and perinatal outcomes. PLoS One. 2014;9(6):e100180 10.1371/journal.pone.0100180 24937406 PMC4061123

[5] Smith GC, Pell JP, Walsh D. Pregnancy complications and maternal risk of ischaemic heart disease: a retrospective cohort study of 129,290 births. Lancet. 2001;357(9273):2002–6. 10.1016/S0140-6736(00)05112-6 11438131

[6] Shah BR, Retnakaran R, Booth GL. Increased risk of cardiovascular disease in young women following gestational diabetes mellitus. Diabetes Care. 2008;31(8):1668–9. 10.2337/dc08-0706 18487472 PMC2494649

[7] Carr DB, Utzschneider KM, Hull RL, Tong J, Wallace TM, Kodama K, et al Gestational diabetes mellitus increases the risk of cardiovascular disease in women with a family history of type 2 diabetes. Diabetes Care. 2006;29(9):2078–83. 10.2337/dc05-2482 16936156

[8] Fraser A, Nelson SM, Macdonald-Wallis C, Cherry L, Butler E, Sattar N, et al Associations of pregnancy complications with calculated cardiovascular disease risk and cardiovascular risk factors in middle age: the Avon Longitudinal Study of Parents and Children. Circulation. 2012;125(11):1367–80. 10.1161/CIRCULATIONAHA.111.044784 22344039 PMC3323835

[9] McDonald SD, Malinowski A, Zhou Q, Yusuf S, Devereaux PJ. Cardiovascular sequelae of preeclampsia/eclampsia: a systematic review and meta-analyses. Am Heart J. 2008;156(5):918–30. 10.1016/j.ahj.2008.06.042 19061708

[10] Gunderson EP, Chiang V, Pletcher MJ, Jacobs DR, Quesenberry CP, Sidney S, et al History of gestational diabetes mellitus and future risk of atherosclerosis in mid-life: the Coronary Artery Risk Development in Young Adults study. J Am Heart Assoc. 2014;3(2):e000490 10.1161/JAHA.113.000490 24622610 PMC4187501

[11] Bellamy L, Casas JP, Hingorani AD, Williams DJ. Pre-eclampsia and risk of cardiovascular disease and cancer in later life: systematic review and meta-analysis. BMJ. 2007;335(7627):974 10.1136/bmj.39335.385301.BE 17975258 PMC2072042

[12] Rodie VA, Freeman DJ, Sattar N, Greer IA. Pre-eclampsia and cardiovascular disease: metabolic syndrome of pregnancy? Atherosclerosis. 2004;175(2):189–202. 10.1016/j.atherosclerosis.2004.01.038 15262174

[13] Romundstad PR, Magnussen EB, Smith GD, Vatten LJ. Hypertension in pregnancy and later cardiovascular risk: common antecedents? Circulation. 2010;122(6):579–84. 10.1161/CIRCULATIONAHA.110.943407 20660802

[14] Qiu C, Williams MA, Leisenring WM, Sorensen TK, Frederick IO, Dempsey JC, et al Family history of hypertension and type 2 diabetes in relation to preeclampsia risk. Hypertension. 2003;41(3):408–13. 10.1161/01.HYP.0000056996.25503.F5 12623936

[15] Geelhoed JJ, Fraser A, Tilling K, Benfield L, Davey Smith G, Sattar N, et al Preeclampsia and gestational hypertension are associated with childhood blood pressure independently of family adiposity measures: the Avon Longitudinal Study of Parents and Children. Circulation. 2010;122(12):1192–9. Epub 2010/09/09. 10.1161/CIRCULATIONAHA.110.936674 20823385 PMC5321267

[16] D'Agostino RB Sr., Vasan RS, Pencina MJ, Wolf PA, Cobain M, Massaro JM, et al General cardiovascular risk profile for use in primary care: the Framingham Heart Study. Circulation. 2008;117(6):743–53. Epub 2008/01/24. 10.1161/CIRCULATIONAHA.107.699579 18212285

[17] Wilson PW, D'Agostino RB, Levy D, Belanger AM, Silbershatz H, Kannel WB. Prediction of coronary heart disease using risk factor categories. Circulation. 1998;97(18):1837–47. 9603539 10.1161/01.cir.97.18.1837

[18] De Sutter J, De Bacquer D, Kotseva K, Sans S, Pyorala K, Wood D, et al Screening of family members of patients with premature coronary heart disease; results from the EUROASPIRE II family survey. Eur Heart J. 2003;24(3):249–57. 12590902 10.1016/s0195-668x(02)00386-x

[19] Lipnick RJ, Buring JE, Hennekens CH, Rosner B, Willett W, Bain C, et al Oral contraceptives and breast cancer. A prospective cohort study. JAMA. 1986;255(1):58–61. Epub 1986/01/03. 3940306

[20] Stampfer MJ, Colditz GA, Willett WC, Manson JE, Rosner B, Speizer FE, et al Postmenopausal estrogen therapy and cardiovascular disease. Ten-year follow-up from the nurses' health study. N Engl J Med. 1991;325(11):756–62. Epub 1991/09/12. 10.1056/NEJM199109123251102 1870648

[21] Michels KB, Rosner BA, Manson JE, Stampfer MJ, Walker AM, Willett WC, et al Prospective study of calcium channel blocker use, cardiovascular disease, and total mortality among hypertensive women: the Nurses' Health Study. Circulation. 1998;97(16):1540–8. Epub 1998/05/21. 9593558 10.1161/01.cir.97.16.1540

[22] Stampfer MJ, Hu FB, Manson JE, Rimm EB, Willett WC. Primary prevention of coronary heart disease in women through diet and lifestyle. N Engl J Med. 2000;343(1):16–22. Epub 2000/07/07. 10.1056/NEJM200007063430103 10882764

[23] Mosca L, Appel LJ, Benjamin EJ, Berra K, Chandra-Strobos N, Fabunmi RP, et al Evidence-based guidelines for cardiovascular disease prevention in women. J Am Coll Cardiol. 2004;43(5):900–21. Epub 2004/03/05. 10.1016/j.jacc.2004.02.001 14998635

[24] Hunt KJ, Schuller KL. The increasing prevalence of diabetes in pregnancy. Obstet Gynecol Clin North Am. 2007;34(2):173–99, vii 10.1016/j.ogc.2007.03.00 17572266 PMC2043158

[25] Ben-Haroush A, Yogev Y, Hod M. Epidemiology of gestational diabetes mellitus and its association with Type 2 diabetes. Diabet Med. 2004;21(2):103–13. 14984444 10.1046/j.1464-5491.2003.00985.x

[26] Anand SS, Islam S, Rosengren A, Franzosi MG, Steyn K, Yusufali AH, et al Risk factors for myocardial infarction in women and men: insights from the INTERHEART study. Eur Heart J. 2008;29(7):932–40. Epub 2008/03/13. 10.1093/eurheartj/ehn018 18334475

[27] Colditz GA, Martin P, Stampfer MJ, Willett WC, Sampson L, Rosner B, et al Validation of questionnaire information on risk factors and disease outcomes in a prospective cohort study of women. Am J Epidemiol. 1986;123(5):894–900. Epub 1986/05/01. 3962971 10.1093/oxfordjournals.aje.a114319

