# Supplementary material for: The Association of Family History of Premature Cardiovascular Disease or Diabetes Mellitus on the Occurrence of Gestational Hypertensive Disease and Diabetes
Source: PLoS One. 2016 Dec 5;11(12):e0167528. doi: 10.1371/journal.pone.0167528 (PMC5137894; doi:10.1371/journal.pone.0167528)
Supplement: S1 File — (DOCX) [file pone.0167528.s001.docx]

**Demographics**

1. Birth date?

Year Month Day

1. Height?

cm

1. Weight? Kg
2. Waist circumference? (If you don’t know exactly, write your pants size)

( inch)

1. Are you married?
2. Currently married (2) Living together without marriage (3) Divorce (4) Bereavement (5) never married

**Family history**

1. Do you have a first-degree male relative (e.g. father, brother) who has suffered a heart attack or stroke before the age of 55, or a first-degree female relative who has suffered ones before the age of 65 ?
2. Yes 2) No
3. Do you have a first-degree relative who has suffered the following diseases?
4. Hypertension 1) Yes 2) No
5. Diabetes mellitus 1) Yes 2) No
6. Dyslipidemia 1) Yes 2) No
7. Stroke 1) Yes 2) No
8. Angina pectoris 1) Yes 2) No
9. Myocardial infarction 1) Yes 2) No

**Obstetric-Gynecologic characteristics**

1. When did you have your first menstruation?
2. <10 y 2) 10 y 3) 11y 4) 12y 5)13y 6) 14y 7)15y 8)16y 9) ≥17 y
3. Is your menstrual cycle regular?
4. Very regular (difference < 1~2 day)
5. Regular (<3~4 day)
6. Regular with larger difference (<5-7 day)
7. Intermittently irregular
8. Almost irregular
9. Unpredictable
10. What is your menstrual cycle length?
11. <21d (2) 21-25d (3) 26-31d (4) 32-39d (5) 40-50d
12. Have you used contraception?
13. Never (2) Used in the past (3) Currently using

4-1. How many months did you use contraception?

(1) 1-4 month (2) 5-9 (3) 10-14 (4) 15-19 (5) 20-25 (6) 26-30

(7) 31-35 (8) ≥ 36

4-2. Which forms of contraception have you used?

1. IUD (2) Foam/Jelly/Sponge (3) Oral contraceptive (4) others

4-3. Which kind oral contraceptives have you used?

1. Myvlar (2) Triquilar (3) Diane (4) Mercilon (5) Minulet (6) Sexcon
2. Have you taken Clomiphene (e.g., Clomid) or Gonadotropin injections (e.g., Gonal-f, Metrodin, Follistim) to induce ovulation?
3. Yes (2) No

5-1. If yes, how long did you take it?

(1) 1 month (2) 2-3 (3) 4-5 (4) 6-11 (5) ≥12

1. Have you ever been pregnant, including miscarriages and induced abortions?
2. Yes (2) No

6-1. Please complete one column of the chart for each of your pregnancies, including miscarriages and induced abortions.

|  | 1st pregnancy | 2nd pregnancy | 3rd pregnancy | 4th pregnancy | 5th pregnancy |
| --- | --- | --- | --- | --- | --- |
| Calendar year in which pregnancy ended? |  |  |  |  |  |
| Outcome of this  pregnancy? | 1)Single live birth  2) Twins/Triplets+  3) Miscarriage/Stillbirth  4) Induced abortion  5) Tubal or Ectopic | 1)Single live birth  2) Twins/Triplets+  3) Miscarriage/Stillbirth  4) Induced abortion  5) Tubal or Ectopic | 1)Single live birth  2) Twins/Triplets+  3) Miscarriage/Stillbirth  4) Induced abortion  5) Tubal or Ectopic | 1)Single live birth  2) Twins/Triplets+  3) Miscarriage/Stillbirth  4) Induced abortion  5) Tubal or Ectopic | 1)Single live birth  2) Twins/Triplets+  3) Miscarriage/Stillbirth  4) Induced abortion  5) Tubal or Ectopic |
| How long did this  pregnancy last? | 1) <8 weeks  2) 8–11 wks  3) 12–19 wks  4) 20–27 wks  5) 28–31 wks  6) 32–36 wks  7) 37–39 wks  8) 40–42 wks  9) 43+ weeks | 1) <8 weeks  2) 8–11 wks  3) 12–19 wks  4) 20–27 wks  5) 28–31 wks  6) 32–36 wks  7) 37–39 wks  8) 40–42 wks  9) 43+ weeks | 1) <8 weeks  2) 8–11 wks  3) 12–19 wks  4) 20–27 wks  5) 28–31 wks  6) 32–36 wks  7) 37–39 wks  8) 40–42 wks  9) 43+ weeks | 1) <8 weeks  2) 8–11 wks  3) 12–19 wks  4) 20–27 wks  5) 28–31 wks  6) 32–36 wks  7) 37–39 wks  8) 40–42 wks  9) 43+ weeks | 1) <8 weeks  2) 8–11 wks  3) 12–19 wks  4) 20–27 wks  5) 28–31 wks  6) 32–36 wks  7) 37–39 wks  8) 40–42 wks  9) 43+ weeks |
| For pregnancies lasting 20+ weeks.... | | | | | |
| Did you have gestational diabetes (GDM)? | 1) Yes 2) No | 1) Yes 2) No | 1) Yes 2) No | 1) Yes 2) No | 1) Yes 2) No |
| If you had GDM, did you have medication? | 1) Yes 2) No | 1) Yes 2) No | 1) Yes 2) No | 1) Yes 2) No | 1) Yes 2) No |
| If you had GDM, was your GDM resolved after delivery? | 1) Yes 2) No | 1) Yes 2) No | 1) Yes 2) No | 1) Yes 2) No | 1) Yes 2) No |
| Did you have gestational hypertension (GHT)? | 1) Yes 2) No | 1) Yes 2) No | 1) Yes 2) No | 1) Yes 2) No | 1) Yes 2) No |
| If you had GHT, did you have medication? | 1) Yes 2) No | 1) Yes 2) No | 1) Yes 2) No | 1) Yes 2) No | 1) Yes 2) No |
| If you had GHT, was your GHT resolved after delivery? | 1) Yes 2) No | 1) Yes 2) No | 1) Yes 2) No | 1) Yes 2) No | 1) Yes 2) No |
| Did you have pre-eclampsia? | 1) Yes 2) No | 1) Yes 2) No | 1) Yes 2) No | 1) Yes 2) No | 1) Yes 2) No |
| Birth weight? | 1) <1.5kg  2) 1.5-2.5kg  3) 2.5-3.5kg  4) 3.5-4.5kg  5) >4.5kg | 1) <1.5kg  2) 1.5-2.5kg  3) 2.5-3.5kg  4) 3.5-4.5kg  5) >4.5kg | 1) <1.5kg  2) 1.5-2.5kg  3) 2.5-3.5kg  4) 3.5-4.5kg  5) >4.5kg | 1) <1.5kg  2) 1.5-2.5kg  3) 2.5-3.5kg  4) 3.5-4.5kg  5) >4.5kg | 1) <1.5kg  2) 1.5-2.5kg  3) 2.5-3.5kg  4) 3.5-4.5kg  5) >4.5kg |
| Type of delivery? | 1) Spontaneous vaginal delivery  2) Induced vaginal delivery  3) C-section | 1) Spontaneous vaginal delivery  2) Induced vaginal delivery  3) C-section | 1) Spontaneous vaginal delivery  2) Induced vaginal delivery  3) C-section | 1) Spontaneous vaginal delivery  2) Induced vaginal delivery  3) C-section | 1) Spontaneous vaginal delivery  2) Induced vaginal delivery  3) C-section |
| Breast feeding duration? | 1) < 1 m  2) 1-3 m  3) 4-6 m  4) 7-11 m  5) >12 m | 1) < 1 m  2) 1-3 m  3) 4-6 m  4) 7-11 m  5) >12 m | 1) < 1 m  2) 1-3 m  3) 4-6 m  4) 7-11 m  5) >12 m | 1) < 1 m  2) 1-3 m  3) 4-6 m  4) 7-11 m  5) >12 m | 1) < 1 m  2) 1-3 m  3) 4-6 m  4) 7-11 m  5) >12 m |
| Weight gain during pregnancy  (kg) |  |  |  |  |  |

7-2. How much did you gain weight at 1 year after the last delivery compared to the weight before the first pregnancy?

1. Equal or Less 2) 1-2kg 3) 3-5kg 4) 6-10kg 5) > 11kg
