# Supplementary material for: The Association of Family History of Premature Cardiovascular Disease or Diabetes Mellitus on the Occurrence of Gestational Hypertensive Disease and Diabetes
Source: PLoS One. 2016 Dec 5;11(12):e0167528. doi: 10.1371/journal.pone.0167528 (PMC5137894; doi:10.1371/journal.pone.0167528)
Supplement: S2 File — (DOCX) [file pone.0167528.s002.docx]

**인구학적 특징**

1. 당신의 생년월일은 언제 입니까?

년 월 일

1. 당신의 키는 얼마입니까?

cm

1. 당신의 몸무게는 얼마입니까?
    Kg
2. 허리 둘레는 얼마입니까?(정확히 모르실 때는 바지 허리 둘레를 입력해주십시오)

( 인치)

1. 귀하는 결혼을 하셨습니까?
2. 기혼 (2) 동거 (3) 이혼 (4) 사별 (5) 미혼 (결혼 한 적이 없음)
3. 본인을 포함한 함께 거주하는 가족수는 몇 명입니까

( ) 명

1. 부양 가족내 연간 총 수입은 어느 정도 입니까?
2. 3천만원 미만 (2) 3천만원-4500만원미만 (3) 4500만원-7500만원미만 (4)7500만원 이상
3. 귀하의 학력은?

□ 3년제 졸  □ 4년제 졸  □ 대학원졸 이상

1. 귀하의 거주지는 어디입니까?

(1) 특별시, 광역시 (2) 시(동) (3) 읍, 면

**가족력**

1. 65세 이전에 심혈관질환 (협심증, 심근경색, 뇌졸중/뇌출혈 등)을 앓은 여성 가족 또는 55세 이전에 심혈관질환을 앓은 남성 가족이 있으십니까?
2. 예 (2) 아니오
3. 연령에 관계 없이 아래와 같은 질병을 가지고 있는 가족이 있으십니까?
4. 고혈압 예 아니오
5. 당뇨 예 아니오
6. 고지혈증 예 아니오
7. 뇌졸중/뇌출혈 예 아니오
8. 협심증 예 아니오
9. 심근경색 예 아니오
10. 유방암 예 아니오
11. 자궁경부암 예 아니오
12. 난소암 예 아니오

**여성학적 특징**

1. 초경은 만으로 몇 세 때 시작하셨습니까?
2. 10살이전 2) 10살 3) 11살 4) 12살 5)13살 6) 14살 7)15살 8)16살 9)17살 이상
3. 월경은 규칙적으로 하십니까?
4. 아주 규칙적이다( 1~2 일 이상 차이가 안남)
5. 규칙적이다 (3~4 일내 차이)
6. 비교적 규칙적이다 (5-7일내)
7. 간혹 불규칙하다
8. 항상 불규칙하다
9. 전혀 예측이 불가능하다
10. 월경은 보통 며칠마다 한 번씩 돌아 옵니까?
11. <21 일 (2) 21-25 (3) 26-31 (4) 32-39 (5) 40-50일마다
12. 피임의 경험이 있습니까?
13. 전혀 없다 (2) 과거사용 (3) 현재 사용 중

5-1. 사용기간

(1) 1-4 개월 (2) 5-9 개월 (3) 10-14 개월 (4) 15-19개월 (5) 20-25 개월 (6) 26-30 개월

(7) 31-35 개월 (8) 36 개월 이상

5-2. 어떤 종류의 피임을 사용했습니까? (다중 선택 가능)

1. 자궁내 장치 (2) 질정 피임약 (3) 경구 피임약 (4) 기타

5-3. 어떤 종류의 경구 피임약을 사용했습니까?(다중 선택 가능)

1. 마이보라 (2) 트리퀼라 (3) 다이안느 (4) 머시론 (5) 미뉴렛 (6) 세스콘
2. 불임치료를 위해 배란촉진제로 clomiphene을 복용하거나 gonadotropin 주사를 맞아 본적이 있습니까?
3. 예 (2) 아니오

6-1.있다면 몇 개월 사용하셨습니까?

(1) 1개월 (2) 2-3개월 (3) 4-5개월 (4) 6-11개월 (5) 12개월 이상

1. 임신경험이 있습니까?
2. 예 (2) 아니오

6-1. 다음은 임신 및 출산에 대한 질문입니다. 본인이 경험했던 모든 임신에 대해 작성해 주십시오.

|  | 첫번째 임신 | 두번째임신 | 세번째임신 | 네번째임신 | 다섯번째임신 |
| --- | --- | --- | --- | --- | --- |
| 임신 년도 |  |  |  |  |  |
| 임신 결과: 다음중택 일 | 1) 단일아 출산  2)쌍생아 및 그 이상 출산  3) 자연유산/사산  4) 인공유산  5) 자궁외임신 | 1) 단일아 출산  2)쌍생아 및 그 이상 출산  3) 자연유산/사산  4) 인공유산  5) 자궁외임신 | 1) 단일아 출산  2)쌍생아 및 그 이상 출산  3) 자연유산/사산  4) 인공유산  5) 자궁외임신 | 1) 단일아 출산  2)쌍생아 및 그 이상 출산  3) 자연유산/사산  4) 인공유산  5) 자궁외임신 | 1) 단일아 출산  2)쌍생아 및 그 이상 출산  3) 자연유산/사산  4) 인공유산  5) 자궁외임신 |
| 임신이 지속된 주수는? | 1) 8주미만  2) 8-11주  3) 12-19주  4) 20-27주  5) 28-31주  6) 32-36주  7) 37-39주  8) 40-42주  9) 43주 이상 | 1) 8주미만  2) 8-11주  3) 12-19주  4) 20-27주  5) 28-31주  6) 32-36주  7) 37-39주  8) 40-42주  9) 43주 이상 | 1) 8주미만  2) 8-11주  3) 12-19주  4) 20-27주  5) 28-31주  6) 32-36주  7) 37-39주  8) 40-42주  9) 43주 이상 | 1) 8주미만  2) 8-11주  3) 12-19주  4) 20-27주  5) 28-31주  6) 32-36주  7) 37-39주  8) 40-42주  9) 43주 이상 | 1) 8주미만  2) 8-11주  3) 12-19주  4) 20-27주  5) 28-31주  6) 32-36주  7) 37-39주  8) 40-42주  9) 43주 이상 |
| 아래는 20주 이상 지속된 임신에 한하여 답변해 주세요. | | | | | |
| 임신성 당뇨가 있었나요? | 1) 예 2) 아니오 | 1) 예 2) 아니오 | 1) 예 2) 아니오 | 1) 예 2) 아니오 | 1) 예 2) 아니오 |
| 임신성 당뇨가 있었던 경우 약물로 치료받았나요? | 1) 예 2) 아니오 | 1) 예 2) 아니오 | 1) 예 2) 아니오 | 1) 예 2) 아니오 | 1) 예 2) 아니오 |
| 임신성 당뇨가 있었던 경우 출산 후 정상화 되었나요? | 1) 예 2) 아니오 | 1) 예 2) 아니오 | 1) 예 2) 아니오 | 1) 예 2) 아니오 | 1) 예 2) 아니오 |
| 임신성 고혈압이 있었나요? | 1) 예 2) 아니오 | 1) 예 2) 아니오 | 1) 예 2) 아니오 | 1) 예 2) 아니오 | 1) 예 2) 아니오 |
| 임신성 고혈압이 있었던 경우 약물로 치료 받았나요? | 1) 예 2) 아니오 | 1) 예 2) 아니오 | 1) 예 2) 아니오 | 1) 예 2) 아니오 | 1) 예 2) 아니오 |
| 임신성 고혈압이 있었던 경우 출산 후 정상화 되었나요? | 1) 예 2) 아니오 | 1) 예 2) 아니오 | 1) 예 2) 아니오 | 1) 예 2) 아니오 | 1) 예 2) 아니오 |
| 전자간증이 있었나요? | 1) 예 2) 아니오 | 1) 예 2) 아니오 | 1) 예 2) 아니오 | 1) 예 2) 아니오 | 1) 예 2) 아니오 |
| 출생 체중은? | 1) 1.5kg 미만  2) 1.5-2.5kg 미만  3) 2.5-3.5kg미만  4) 3.5-4.5kg 미만  5) 4.5kg이상 | 1) 1.5kg 미만  2) 1.5-2.5kg 미만  3) 2.5-3.5kg미만  4) 3.5-4.5kg 미만  5) 4.5kg이상 | 1) 1.5kg 미만  2) 1.5-2.5kg 미만  3) 2.5-3.5kg미만  4) 3.5-4.5kg 미만  5) 4.5kg이상 | 1) 1.5kg 미만  2) 1.5-2.5kg 미만  3) 2.5-3.5kg미만  4) 3.5-4.5kg 미만  5) 4.5kg이상 | 1) 1.5kg 미만  2) 1.5-2.5kg 미만  3) 2.5-3.5kg미만  4) 3.5-4.5kg 미만  5) 4.5kg이상 |
| 출산 방법은? | 1) 자연분만  2) 유도질식분만  3) 제왕절개 | 1) 자연분만  2) 유도질식분만  3) 제왕절개 | 1) 자연분만  2) 유도질식분만  3) 제왕절개 | 1) 자연분만  2) 유도질식분만  3) 제왕절개 | 1) 자연분만  2) 유도질식분만  3) 제왕절개 |
| 모유수유 기간은? | 1) 1개월 미만  2) 1-3개월  3) 4-6개월  4) 7-11개월  5) 12개월 이상 | 1) 1개월 미만  2) 1-3개월  3) 4-6개월  4) 7-11개월  5) 12개월 이상 | 1) 1개월 미만  2) 1-3개월  3) 4-6개월  4) 7-11개월  5) 12개월 이상 | 1) 1개월 미만  2) 1-3개월  3) 4-6개월  4) 7-11개월  5) 12개월 이상 | 1) 1개월 미만  2) 1-3개월  3) 4-6개월  4) 7-11개월  5) 12개월 이상 |
| 임신기간중의 체중 증가는?  (kg, 주관식) |  |  |  |  |  |

7-2. 마지막 출산 1년 후 체중이 최초 임신 전과 비교하여 어느정도 증가되었습니까?

1. 같거나 줄었다 2) 1-2kg 3) 3-5kg 4) 6-10kg 5) 11kg 이상
