# Supplementary material for: The Association of Family History of Premature Cardiovascular Disease or Diabetes Mellitus on the Occurrence of Gestational Hypertensive Disease and Diabetes
Source: PLoS One. 2016 Dec 5;11(12):e0167528. doi: 10.1371/journal.pone.0167528 (PMC5137894; doi:10.1371/journal.pone.0167528)
Supplement: S1 Table — (DOCX) [file pone.0167528.s003.docx]

**Supplementary table 1. Comparison of the subjects with GHD and GDM in the same pregnancy to the others**

|  | No gestational disease  (n = 3,553) | GHD only  (n = 222) | GDM only  (n = 98) | GHD & GDM in the same pregnancy (n = 22) | *p* |
| --- | --- | --- | --- | --- | --- |
| Age at the survey (years) | 40.3 ± 8.0 | 40.6 ± 7.9 | 38.9 ± 6.0 | 44.3 ± 7.2 | 0.035 |
| BMI (kg/m^2^) at the survey | 21.8 ± 2.5 | 22.5 ± 2.6 | 22.1 ± 2.5 | 22.1 ± 1.9 | 0.001 |
| Age at the pregnancy | 28.6 ± 3.3 | 30.1 ± 3.4 | 31.2 ± 3.0 | 30.4 ± 3.9 | <0.001 |
| Menarche ≤ 12years | 541 (15.2%) | 38 (17.1%) | 16 (16.3%) | 3 (13.6%) | 0.874 |
| Menstrual regularity (<4 days) | 2276 (64.1%) | 149 (67.1%) | 51 (52.0%) | 16 (72.7%) | 0.051 |
| Menstrual cycle (<30 days) | 2753 (77.5%) | 180 (81.1%) | 64 (65.3%) | 18 (81.8%) | 0.017 |
| Use of oral contraceptive pill | 343 (9.7%) | 18 (8.1%) | 10 (10.2%) | 2 (9.1%) | 0.890 |
| Infertility therapy | 260 (7.3%) | 20 (9.0%) | 13 (13.3%) | 2 (9.1%) | 0.133 |
| Premature CVD* | 842 (23.7%) | 76 (34.2%) | 29 (29.6%) | 8 (36.4%) | 0.001 |
| Hypertension | 2,026 (57.0%) | 144 (64.9%) | 60 (61.2%) | 15 (68.2%) | 0.078 |
| Diabetes mellitus | 1,011(28.5%) | 65 (29.3%) | 53 (54.1%) | 9 (40.9%) | <0.001 |
| Dyslipidemia | 1,097 (30.9%) | 83 (37.4%) | 37 (37.8%) | 9 (40.9%) | 0.075 |
| Stroke | 535 (15.1%) | 49 (22.1%) | 18 (18.4%) | 6 (27.3%) | 0.013 |
| Angina pectoris | 313 (8.8%) | 20 (9.0%) | 16 (16.3%) | 3 (13.6%) | 0.068 |
| Myocardial infarction | 177 (5.0%) | 12 (5.4%) | 4 (4.1%) | 0 (0%) | 0.704 |
